# *Salmonella* produces sulfide to compete with *Escherichia coli* in the gut lumen

**DOI:** 10.1073/pnas.2504095122

**Published:** 2025-09-12

**Authors:** Anaïs B. Larabi, Connor R. Tiffany, Hugo L. P. Masson, Henry Nguyen, Eli J. Bejarano, Megan J. Liou, Lauren C. Radlinski, Aurore M. Demars, Renée M. Tsolis, Andreas J. Bäumler

**Affiliations:** ^a^Department of Medical Microbiology and Immunology, School of Medicine, University of California, Davis, CA 95616

**Keywords:** *Salmonella*, microbiota, desulfovibrio, *Escherichia coli*, colonization resistance

## Abstract

Hydrogen sulfide production is a characteristic that distinguishes *Salmonella* serovars from closely related species, such as *Escherichia coli*, but its biological significance remains obscure. Here, we show that PhsABC and AsrABC-mediated hydrogen sulfide production by *Salmonella enterica* serovar (*S.*) Typhimurium was linked to an increased abundance of *Deltaproteobacteria* and inhibition of cytochrome *bd* oxidase-mediated aerobic respiration of *Escherichia coli* in the murine large intestine. Functional *phsABC* and *asrABC* operons provided a growth benefit to *S.* Typhimurium in the cecum of *Enterobacterales-*free conventional mice only upon inoculation with commensal *E. coli.* In gnotobiotic mice engrafted with a defined community of 17 human *Clostridia* isolates, *S.* Typhimurium infection inhibited cytochrome *bd* oxidase-mediated aerobic respiration in *E. coli* only in the presence of *Desulfovibrio piger*, a sulfide-producing representative of the *Deltaproteobacteria.* A *S.* Typhimurium strain deficient for hydrogen sulfide production (*phsA asrA* mutant) did not inhibit cytochrome *bd* oxidase-mediated aerobic respiration in *E. coli*, even when *D. piger* was present. Collectively, these data suggest that the *phsABC* and *asrABC* operons of *Salmonella* serovars provide a benefit during competition with closely related bacteria, such as *E. coli*, by inhibiting cytochrome *bd* oxidase-mediated aerobic respiration of the commensal.

## Significance

The microbiota is thought to confer resistance against *Salmonella* colonization by consuming resources that overlap with those used by the pathogen. Colonization resistance increases with community diversity, which rests critically upon the presence of close relatives, such as *Escherichia coli.* Here, we show that *Salmonella enterica* serovar (*S.*) Typhimurium gains an edge during this competition by producing hydrogen sulfide to limit aerobic respiration of *E. coli*. Thus, hydrogen sulfide production, a biochemical feature that has been used for a century to distinguish *Salmonella* serovars from *E. coli*, turns out to be part of a business plan that enables the pathogen to break microbiota-mediated colonization resistance during infection.

*Salmonella* serovars are pathogenic members of the *Enterobacterales* that can be differentiated from closely related *Enterobacterales* species, such as *Escherichia coli*, by their ability to produce black precipitates on bismuth sulfite agar (1, 2) or in triple sugar iron agar slants (3). Enzymes encoded by the *Salmonella enterica* serovar (*S.*) Typhimurium *phsABC* operon catalyze reduction of thiosulfate (S_2_O_4_^2−^) to hydrogen sulfide (H_2_S) (anaerobic thiosulfate respiration) (4) leading to the formation of black ferrous sulfide (FeS) precipitates in triple sugar iron agar slants (3). Enzymes encoded by the *S.* Typhimurium *asrABC* operon catalyze reduction of sulfite (SO_3_^2−^) to hydrogen sulfide (H_2_S) (anaerobic sulfite respiration) (5, 6), which leads to the formation of black bismuth sulfide (Bi_2_S_3_) precipitates on bismuth sulfite agar (1, 2). In contrast, growth of *E. coli* does not result in the formation of black precipitates on either of these media.

Expression of the *phsABC* and *asrABC* operons is induced during growth of *S.* Typhimurium in the lumen of the murine large intestine (7). Furthermore, hydrogen sulfide production is conserved among all *Salmonella* serovars associated with gastrointestinal disease, such as *S.* Typhimurium, but varies in serovars associated with human extraintestinal disease, including *S.* Typhi, *S.* Paratyphi A, and *S.* Sendai (8). These associations raise the possibility that sulfide production is under selection during gastrointestinal disease (9), but the underlying mechanisms remain elusive.

Bacterial sulfur metabolism in the healthy intestine is linked to the deconjugation of primary bile acids, which releases taurine (10). In the large intestine, sulfate-reducing bacteria of the class *Deltaproteobacteria* convert taurine to ethanolamine and hydrogen sulfide (11, 12). Hydrogen sulfide can inhibit cytochrome *c* oxidase in the mitochondrial respiratory chain (13). To protect the epithelial surface, the mitochondrial sulfide quinone oxidoreductase oxidizes hydrogen sulfide to the nontoxic compound thiosulfate (S_2_O_3_^2−^) (14–16). Reactive oxygen species generated during *S.* Typhimurium-induced intestinal inflammation oxidize thiosulfate to tetrathionate (S_4_O_6_^2−^), thereby generating an electron acceptor for anaerobic respiration that drives luminal pathogen growth (17).

Infection with *Yersinia pseudotuberculosis*, a pathogenic member of the *Enterobacterales*, increases the flow of bile into the large intestine (18). The resulting rise in taurine availability in the large intestine heightens *Deltaproteobacteria-*mediated production of hydrogen sulfide, thereby inhibiting cytochrome *bd* oxidase-mediated aerobic respiration of *Citrobacter rodentium*, another member of the *Enterobacterales* (18). Chemically induced colitis or antibiotic treatment elevates oxygen availability in the large intestine, which fuels growth of *E. coli* through cytochrome *bd* oxidase-mediated aerobic respiration (19, 20). Oxygen availability in the cecal lumen also becomes elevated during *S.* Typhimurium infection (21, 22). Here, we determined whether *phsABC* and *asrABC*-mediated hydrogen sulfide production by *S.* Typhimurium enables the pathogen to limit growth of close competitors, such as *E. coli*, by limiting their growth by aerobic respiration.

## Results

### *S.* Typhimurium Infection Does Not Fuel *E. coli* Growth by Aerobic Respiration.

During infection of mice, *S.* Typhimurium uses two type III secretion systems (T3SS-1 and T3SS-2) to trigger inflammation in the cecum (23, 24). The host inflammatory response increases diffusion of oxygen into the cecal lumen to fuel growth of *S.* Typhimurium by aerobic respiration (21, 22). To determine whether *E. coli* can take advantage of elevated oxygen availability during *S.* Typhimurium infection, we used genetically resistant CBA mice from Jackson laboratories (CBA/J mice), which do not carry endogenous *Enterobacterales*, because the vendor screens against the presence of this taxon in its special pathogen-free procedures (25). Since the ecological niche of *Enterobacterales* remains vacant in CBA/J mice, engraftment with *E. coli* strains provides an opportunity for precision editing of the microbiota (26).

**Fig. 1.**
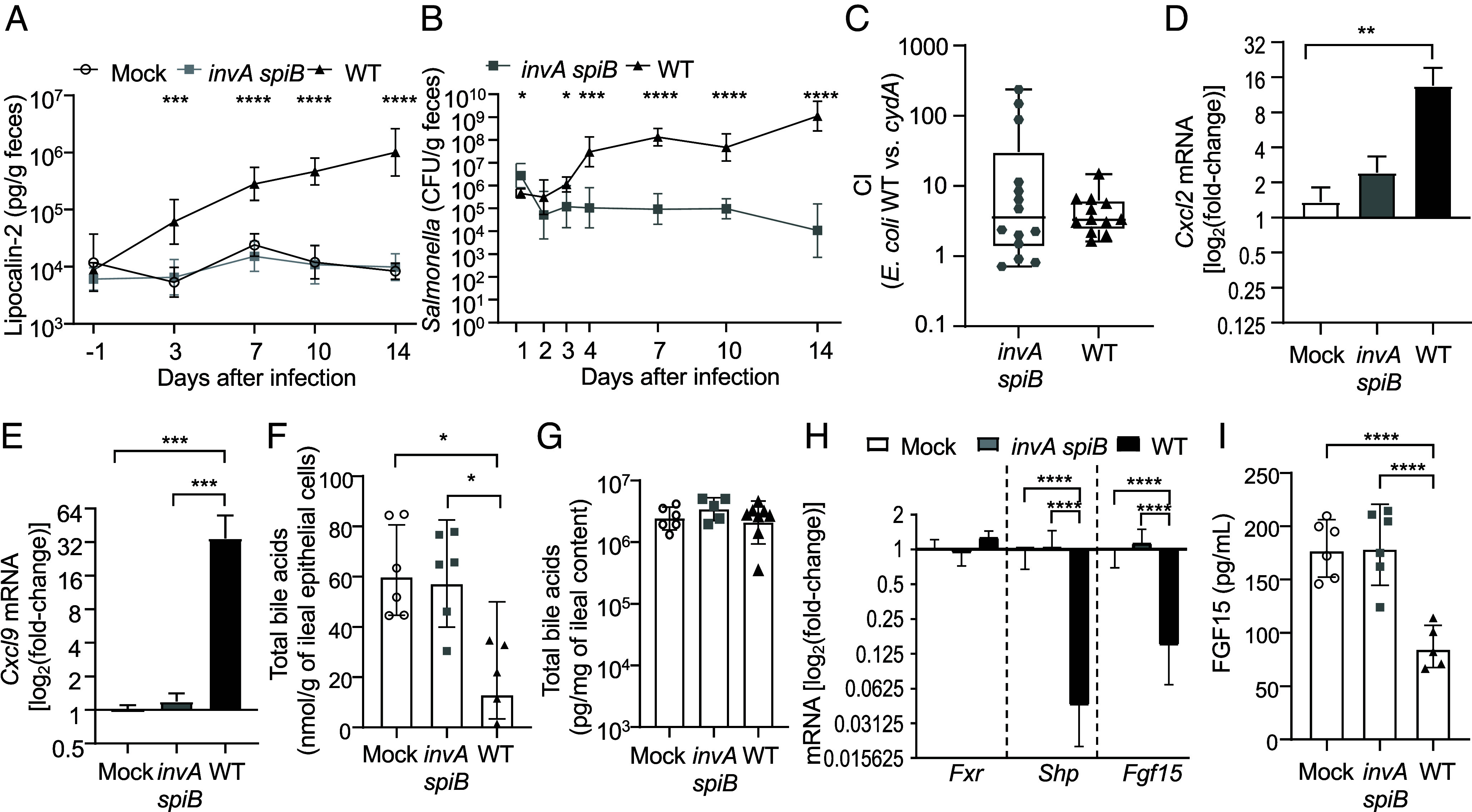
*S.* Typhimurium-induced inflammation triggers bile acid malabsorption in the ileum. (*A* and *B* and *D*–*H*) Groups of antibiotic-naïve, *Enterobacterales-*free CBA/J mice were mock infected (mock, N = 6) or infected with 10^9^ CFU of an avirulent *S.* Typhimurium *invA spiB* mutant (*invA spiB*, N = 6) or the *S.* Typhimurium wild type (WT, N = 5). (*A* and *B*) At the indicated timepoints, feces were collected to determine fecal lipocalin-2 levels (*A*) and *S.* Typhimurium colonization by enumerating CFU (*B*). (*C*) CBA/J mice infected with 10^9^ CFU of an avirulent *S.* Typhimurium *invA spiB* mutant (*invA spiB*, N = 14) or the *S.* Typhimurium wild type (WT, N = 12). Ten days later, mice were inoculated with a 1:1 mixture of *E. coli* strain Nissle 1917 (WT) and an isogenic *cydA* mutant (*cydA*). The CI in feces was determined 1 d later. The box plots represent the first to third quartiles, and the line indicates the median value. (*D* and *E*) RNA was isolated from Ileal Peyer’s patches collected 14 d after infection and transcript levels of *Cxcl2* (*D*) and *Cxcl9* (*E*) determined by quantitative real-time PCR. (*F* and *H*) Ileal epithelial cells were collected 14 d after infection. (*F* and *G*) Total bile acid concentrations in lysates of ileal epithelial cells (*F*) or in ileal contents (*G*) were determined using a colorimetric assay. (*H*) Fold-change in transcript levels of the indicated genes compared to mock-infected mice were determined in RNA isolated from ileal epithelial cells by quantitative real-time PCR. (*I*) Serum concentrations of FGF15 were measured by ELISA 14 d after infection. (*A*–*I*) Data represent geometric means ± geometric SD. The unpaired *t* test with Welch’s correction (*B* and *C*) or ordinary one-way ANOVA with the Tukey post-test (*A* and *D*–*I*) was performed on logarithmically transformed values. ^∗^*P* < 0.05; ^∗∗^*P* < 0.01; ^∗∗∗^*P* < 0.001; ^∗∗∗∗^*P* < 0.0001.

CBA/J mice, which were culture confirmed to be *Enterobacterales-*free, were mock infected or infected *via* oral gavage with 10^9^ colony-forming units (CFU) of the *S.* Typhimurium wild type (strain AJB715, a virulent derivative of isolate ATCC14028 that is marked with an antibiotic resistance cassette) (27) or an avirulent *S.* Typhimurium *invA spiB* mutant. Mutations in *invA* and *spiB* result in a functional inactivation of T3SS-1 and T3SS-2, respectively (28), thereby rendering *S.* Typhimurium unable to trigger intestinal inflammation (23, 24). The concentration of fecal lipocalin-2, a marker of intestinal inflammation, was significantly elevated in mice starting 3 d after infection with the *S.* Typhimurium wild type compared to mice infected with an avirulent *S.* Typhimurium *invA spiB* mutant (Fig. 1*A*). After an initial decrease, fecal pathogen burdens increased on days 3 and 4 after infection with the *S.* Typhimurium wild type, but not after infection with an avirulent *invA spiB* mutant (Fig. 1*B*). Ten days after *S.* Typhimurium infection, mice were inoculated with a 1:1 mixture of *E. coli* strain Nissle 1917 and an isogenic *cydA* mutant, which lacks cytochrome *bd* oxidase, and the competitive index (CI) was determined 1 d later. Cytochrome *bd* oxidase conferred a fitness advantage in mice infected with virulent *S.* Typhimurium, but this fitness advantage was not reduced in mice infected with an avirulent *S.* Typhimurium mutant (Fig. 1*C*). This result was unexpected, since a T3SS-1/2-mediated increase in oxygen availability during *S.* Typhimurium infection (21, 22) is predicted to enhance cytochrome *bd* oxidase-mediated growth of *E. coli* (19, 20). Collectively, these findings suggested that *S.* Typhimurium prevents *E. coli* from taking advantage of increased oxygen availability during infection, but the underlying mechanism remained elusive.

### *S.* Typhimurium Infection Induces Malabsorption of Bile Acids in the Ileum.

Previous work shows that *Y. pseudotuberculosis* infection increases the flow of bile into the large intestine to increase *Deltaproteobacteria* abundance, thereby inhibiting cytochrome *bd* oxidase-mediated aerobic respiration of *C. rodentium* (18). Like *Y. pseudotuberculosis*, *S.* Typhimurium uses its virulence factors (i.e., T3SS-1 and T3SS-2) to enter and survive in ileal Peyer’s patches (29–31). To determine whether inflammation of ileal Peyer’s patches is observed in our model, transcript levels of the proinflammatory genes *Cxcl2* (encoding C-X-C motif chemokine ligand 2) and *Cxcl9* were determined in messenger RNA isolated from ileal Peyer’s patches. *Cxcl2* and *Cxcl9* expression was significantly elevated in ileal Peyer’s patches of mice infected with the *S.* Typhimurium wild type compared mice infected with an avirulent *invA spiB* mutant or mock-infected mice (Fig. 1 *D* and *E*).

In patients with Crohn’s disease, ileitis induces malabsorption of bile acids to increase their flow into the large intestine (32). Since *S.* Typhimurium-induced ileitis triggers malabsorption of amino acids (33), we wanted to determine whether the pathogen also impaired bile acid absorption. Consistent with bile acid malabsorption, ileal epithelial cells from mice infected with the *S.* Typhimurium wild type exhibited a significantly reduced intracellular bile acid concentration compared to cells from mice infected with an avirulent *S.* Typhimurium *invA spiB* mutant or uninfected mice (Fig. 1*F*), although bile acid concentrations in ileal contents were similar in all groups (Fig. 1*G*). In enterocytes, primary bile acids bind to the nuclear receptor farnesoid X receptor (FXR), which plays a critical role in regulating bile acid synthesis, transport, secretion, and absorption (reviewed in (10)). Upon bile acid absorption, FXR dimerizes with retinoid X receptor (RXR) to activate the transcription of several genes involved in bile acid transport and synthesis, such as the small heterodimer partner gene (*Shp*), and the fibroblast growth factor 15 gene (*Fgf15*). FGF15 is secreted to the portal circulation where it signals the liver to reduce de novo bile acid synthesis via a negative feedback loop (reviewed in ref. 10). In mice infected with the *S.* Typhimurium wild type, we observed reduced levels of *Shp* and *Fgf15* transcripts in RNA isolated from ileal epithelial cells (Fig. 1*H*), and decreased serum levels of FGF15 protein (Fig. 1*I*) compared to mock-infected or *S.* Typhimurium *invA spiB*-infected mice. These observations were consistent with our hypothesis that ileal inflammation induced during *S.* Typhimurium infection is associated with impaired bile acid absorption and a disruption of the FGF15-mediated negative feedback loop.

### *Salmonella*-Induces a Rise in *Deltaproteobacteria* Abundance Despite Lowering the Concentration of Luminal Bile Acids in the Cecum.

**Fig. 2.**
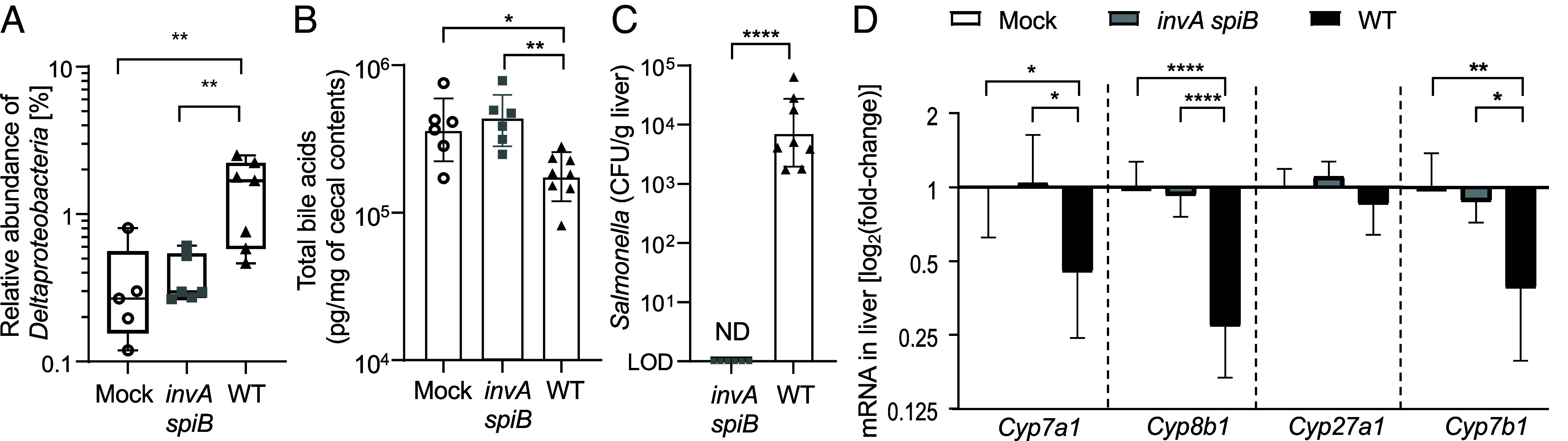
*Deltaproteobacteria* increase in relative abundance during *S.* Typhimurium-induced colitis despite reduced cecal bile acid concentrations. Groups of CBA/J mice were mock infected (mock, N = 5 to 6) or infected with 10^9^ CFU of an avirulent *S.* Typhimurium *invA spiB* mutant (*invA spiB*, N = 6) or the *S.* Typhimurium wild type (WT, N = 5 to 8). (*A*) Cecal contents were isolated 15 d after infection and the relative abundance of *Deltaproteobacteria* compared to total *Eubacteria* was determined using quantitative real-time PCR. The box plots represent the first to third quartiles, and the line indicates the median value. (*B*) Total bile acid concentrations were measured in cecal contents collected 12 d after infection using liquid chromatography/mass spectrometry (LC–MS). (*C*) *S*. Typhimurium colonization of the liver was determined by enumerating CFU 12 d after infection. (*D*) Fold-change compared to mock-infected mice of *Cyp7a1, Cyp8b1, Cyp27a1, and Cyp7b1* transcript levels were determined by quantitative real-time PCR in RNA isolated from the liver collected 12 d after infection. Ordinary one-way ANOVA with Tuckey post-test was performed on logarithmically transformed values. ^∗^*P* < 0.05; ^∗∗^*P* < 0.01; ^∗∗∗^*P* < 0.001; ND, not detected.

Elevated bile acid levels in the large intestine can promote the expansion of *Deltaproteobacteria* (11, 18), a class of obligately anaerobic bacteria that are credited with hydrogen sulfide production in the large intestine. We thus determined the abundance of *Deltaproteobacteria* using quantitative real-time PCR using class-specific primers. Relative to the total abundance of *Eubacteria*, the abundance of *Deltaproteobacteria* was significantly elevated in cecal contents of mice infected with the *S.* Typhimurium wild type compared to mock-infected mice or mice infected with an avirulent *invA spiB* mutant (Fig. 2*A*). Next, we investigated whether the *S.* Typhimurium-induced increase in *Deltaproteobacteria* abundance was linked to elevated bile acid concentrations. Surprisingly, mice infected with the *S.* Typhimurium wild type exhibited lower bile acid concentration in their ceca compared to mock-infected mice or mice infected with an avirulent *invA spiB* mutant (Fig. 2*B*).

A possible explanation for our observation was that exposure to lipopolysaccharide (LPS) reduces the liver content of cytochrome P450 (34), a key enzyme for primary bile acid synthesis. *S.* Typhimurium infection triggers a prominent toll-like receptor 4 (TLR4) and caspase-11-mediated response against LPS in the liver and spleen of mice (35, 36). A high bacterial burden was detected in the liver of mice infected with the *S.* Typhimurium wild type but not in mice infected with an avirulent *invA spiB* mutant (Fig. 2*C*). We thus determined whether infection with the *S.* Typhimurium wild type decreased expression of genes involved in bile acid synthesis, including *Cyp7a1*, *Cyp8b1*, *Cyp27a1*, and *Cyp7b1*. CYP7A1 initiates the classic bile acid pathway, CYP8B1 plays a role in cholic acid production, whereas CYP27A1 and CYP7B1 are involved in the alternative bile acid synthesis pathway. Notably, expression of *Cyp7a1*, *Cyp8b1*, and *Cyp7b1* was significantly reduced in the liver of mice infected with the *S.* Typhimurium wild type compared to the other groups (Fig. 2*D*). Thus, although *S.* Typhimurium infection triggered ileal bile acid malabsorption (Fig. 1 *F*, *H*, and *I*), it reduced bile acid concentrations in the large intestine (Fig. 2*B*), presumably because pathogen dissemination to the liver (Fig. 2*C*) reduced bile acid synthesis (Fig. 2*D*). However, the latter was not further investigated.

Collectively, our data suggested that a *S.* Typhimurium-induced increase in *Deltaproteobacteria* abundance in cecal contents was linked to virulence-factor induced colitis but could not be explained by elevated levels of luminal bile acids.

### Sulfide Production Links *Salmonella* Infection to *Deltaproteobacteria* Expansion.

**Fig. 3.**
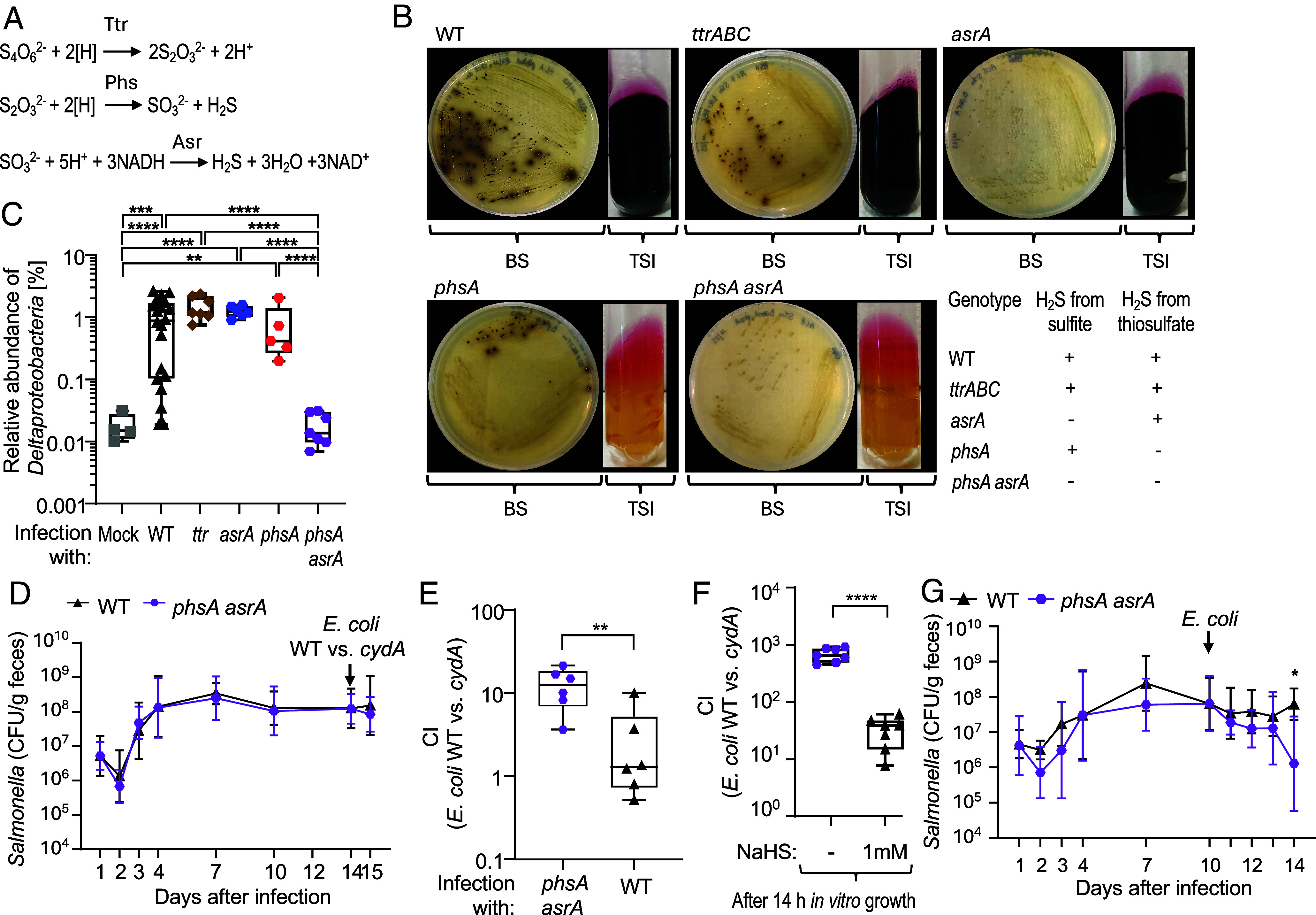
The ability of *S.* Typhimurium to produce hydrogen sulfide provides a benefit during competition with *E. coli.* (*A*) Biochemical reactions catalyzed by enzymes encoded by the *ttr*, *phs,* and *asr* operons of *S.* Typhimurium. S_4_O_6_^2−^, tetrathionate; S_2_O_3_^2−^, thiosulfate; SO_3_^2−^, sulfite; H_2_S, hydrogen sulfide; H_2_O, water; [H], electron; NADH and NAD^+^, reduced and oxidized forms of nicotinamide adenine dinucleotide. (*B*) The images show growth at 37 °C for 24 h of the indicated *S.* Typhimurium strains on bismuth sulfite agar plates (BS) and triple sugar iron agar slants (TSI). *S.* Typhimurium wild type, WT; *ttrABC* mutant, *ttrABC*; *asrA* mutant; *asrA*; *phsA* mutant; *phsA*; *phsA asrA* mutant; *phsA asrA*. The ability (+) or inability (−) of each strain to produce hydrogen sulfide (H_2_S) from sulfite or thiosulfate is indicated at the bottom right. (*C*) Groups of CBA/J mice were infected with 10^9^ CFU of the indicated *S.* Typhimurium wild type (WT, N = 13), a *ttrABC* mutant (*ttrABC*, N = 7), an *asrA* mutant (*asrA*, N = 6), a *phsA* mutant (*phsA*, N = 5), or a *phsA asrA* mutant (*phsA asrA*, N = 7). The relative abundance of *Deltaproteobacteria* compared to total *Eubacteria* was determined by quantitative real-time PCR using genomic DNA isolated from cecal contents collected 14 d after infection. (*D* and *E*) Groups of CBA/J mice were infected with 10^9^ CFU of a *S.* Typhimurium *phsA asrA* mutant (*phsA asrA*, N = 6) or the *S.* Typhimurium wild-type (WT, N = 6). Fourteen days later, mice were infected with 10^9^ CFU of a 1:1 mixture of *E. coli* Nissle 1917 (WT) and an isogenic *cydA* mutant (*cydA*). (*D*) Feces were collected at the indicated timepoints to determine *S.* Typhimurium CFU. (*E*) The CI of *E. coli* wild type vs. *cydA* mutant was determined in the cecum 24 h after inoculation with *E. coli*. (*F*) LB broth containing 1% calcium carbonate was inoculated with 10^3^ CFU of a 1:1 mixture of the *E. coli* Nissle 1917 wild type and an isogenic c*ydA* mutant, in presence or absence of 1 mM sodium hydrosulfide (NaHS). The CI was determined after 14 h of standing culture under atmospheric conditions at 37 °C. (*G*) Groups of CBA/J mice were infected with 10^9^ CFU of a *S.* Typhimurium *phsA asrA* mutant (N = 7) or the *S.* Typhimurium wild-type (N = 5). Ten days later, mice were infected with 10^9^ CFU of *E. coli* Nissle 1917. The graph shows *S.* Typhimurium CFU recovered from feces at the indicated time points after infection. (*C* and *E*) The box plots represent the first to third quartiles, and the line indicates the median value. (*C*–*G*) Data represent geometric means ± geometric SD. The unpaired *t* test with Welch’s correction (*D*–*G*) or ordinary one-way ANOVA with the Tukey post-test (*C*) was performed on logarithmically transformed values. ^*^P < 0.05; ^∗∗^*P* < 0.01; ^∗∗∗∗^*P* < 0.0001.

In addition to taurine, *Deltaproteobacteria* can utilize sulfite and thiosulfate as terminal electron acceptors (37). We thus hypothesized that the ability of *S.* Typhimurium to produce these sulfur-containing compounds might explain the observation that infection with the pathogen is accompanied by an increased *Deltaproteobacteria* abundance. Enzymes encoded by the *S.* Typhimurium *ttrABC* operon reduce tetrathionate (S_4_O_6_^2−^) to thiosulfate (S_2_O_3_^2−^). Thiosulfate is further reduced to sulfite (SO_3_^2−^) and hydrogen sulfide by enzymes encoded in the *phsABC* operon (4). Finally, *S.* Typhimurium reduces sulfite to hydrogen sulfide and water using enzymes encoded by the *asrABC* operon (5, 6) (Fig. 3*A*). Reduction of thiosulfate to hydrogen sulfide in thiosulfate triple sugar agar slants and reduction of sulfite to hydrogen sulfide on bismuth sulfite agar is indicated by black precipitates of ferrous sulfide or bismuth sulfide, respectively (1–3). Deletion of the *ttrABC* genes did not prevent the formation of black sulfide precipitates in either medium. As expected, genetic inactivation of *phsA* prevented the formation of black ferrous sulfide precipitates in thiosulfate triple sugar agar slants, whereas deletion of *asrA* prevented formation of black colonies on bismuth sulfite agar. However, only a *phsA asrA* mutant was unable to produce hydrogen sulfide from either source (Fig. 3*B*).

To investigate whether the *ttr*, *phs*, and/or *asr* operons are required for increasing the abundance of *Deltaproteobacteria* during *S.* Typhimurium infection, CBA/J mice were infected with 10^9^ CFU of the *S.* Typhimurium wild type, a *ttrABC* mutant, an *asrA* mutant, a *phsA* mutant, or a *phsA asrA* mutant. The relative abundance of *Deltaproteobacteria* relative to total *Eubacteria* was determined in DNA isolated from the cecal contents collected 14 d after infection. Contrary to our hypothesis, the relative *Deltaproteobacteria* abundance was similar in cecal contents of mice infected with the *S.* Typhimurium wild type compared to cecal contents of mice infected with mutants unable to produce thiosulfate (*ttrABC* mutant), or sulfite (*phsA* mutant). However, the relative *Deltaproteobacteria* abundance was significantly reduced in cecal contents of mice infected with a *S.* Typhimurium mutant that was unable to produce hydrogen sulfide (*phsA asrA* mutant) (Fig. 3*C*). These data suggested that an elevated *Deltaproteobacteria* abundance during *S.* Typhimurium infection required virulence factors (Fig. 2*A*) and hydrogen sulfide production by the pathogen (Fig. 3*B*).

### Hydrogen Sulfide Production by *Salmonella* Reduces *cydA*-Mediated Growth of *E. coli*.

Next, we wanted to determine whether an inability of *S.* Typhimurium to produce hydrogen sulfide would enable *E. coli* to use cytochrome *bd* oxidase to take advantage of increased oxygen availability during infection. To test this idea, CBA/J mice were infected with either the *S.* Typhimurium wild type or a *phsA asrA* mutant, and bacterial burden in the feces was monitored (Fig. 3*D*). Fourteen days after infection, mice were orally inoculated with 10^9^ CFU of a 1:1 mixture of the *E. coli* Nissle 1917 wild type and an isogenic c*ydA* mutant. The CI between these two *E. coli* strains was assessed in the cecum 24 h later. Notably, cytochrome *bd* oxidase-mediated aerobic respiration conferred a greater fitness advantage upon *E. coli* in mice infected with a *S.* Typhimurium *phsA asrA* mutant compared to mice infected with the *S.* Typhimurium wild type (Fig. 3*E*). These results suggested that hydrogen sulfide production by *S.* Typhimurium directly or indirectly reduced the ability of *E. coli* to use *cydA* for driving its growth by aerobic respiration. To directly test whether sulfide reduces *cydA*-mediated growth of *E. coli*, a 1:1 mixture of the *E. coli* Nissle 1917 wild type and an isogenic c*ydA* mutant was cultured in the presence or absence of 1 mM of sodium hydrosulfide (NaHS) under microaerophilic conditions. Consistent with our hypothesis, sulfide reduced the fitness advantage conferred upon *E. coli* by cytochrome *bd* oxidase-mediated aerobic respiration (Fig. 3*F*).

The contribution of commensal *Enterobacterales*, such as *E. coli*, to colonization resistance against pathogenic members of the *Enterobacterales*, such as *S.* Typhimurium, is disproportionally large, because these close relatives exhibit extensive overlap in their nutrient requirements (38). We thus determined whether inhibition of cytochrome *bd* oxidase-mediated aerobic respiration in *E. coli* would provide a growth advantage to the pathogen. The *S.* Typhimurium wild type and a *S.* Typhimurium *phsA asrA* mutant colonized *Enterobacterales-*free CBA/J mice at similar levels (Fig. 3*D*), suggesting that hydrogen sulfide production did not provide a growth benefit in the absence of *E. coli*. In contrast, when CBA/J mice were infected with the *S.* Typhimurium wild type or a *phsA asrA* mutant, inoculation with *E. coli* reduced the fecal burden of the *phsA asrA* mutant compared to the *S.* Typhimurium wild type by 4 d after inoculation (Fig. 3*G*). These data suggested that *phsABC* and *asrABC*-mediated sulfide production conferred a fitness advantage upon *S.* Typhimurium only in the presence of a close competitor, such as *E. coli.*

### Deltaproteobacteria Are Essential for *Salmonella* to Inhibit *cydA*-Mediated Growth of *E. coli*.

**Fig. 4.**
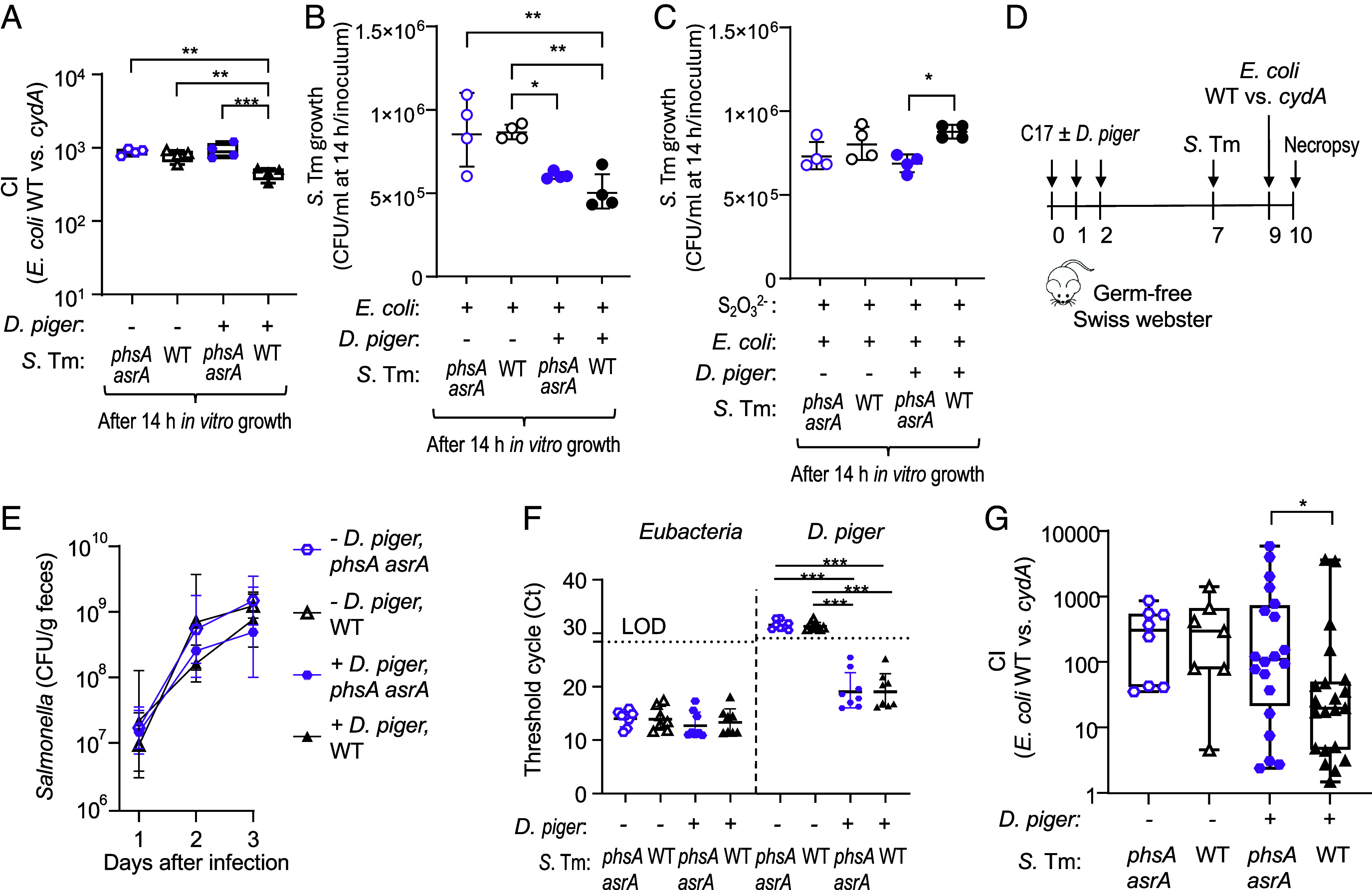
Inhibition of aerobic respiration in *E. coli* requires the presence of *D. piger* and hydrogen sulfide production by *S.* Typhimurium. (*A*–*C*) The indicated *S.* Typhimurium (*S.* Tm) strains [wild type (WT) or *phsA asrA* mutant], the *E. coli* Nissle 1917 wild type (*E. coli* WT) and a *E. coli cydA* mutant were cultured in the presence or absence of *D. piger* in trypticase soy broth supplemented with sheep blood and calcium carbonate. (*A*) The CI of *E. coli* WT versus *cydA* mutant was assessed after 14 h of incubation. (*B* and *C*) Growth of *S.* Typhimurium strains was determined by dividing CFU/mL recovered after 14 h of incubation by CFU/ml in the inoculum. (*C*) Growth was performed in the presence of 3% thiosulfate (S_2_O_3_^2−^). (D–G) Groups of germ-free Swiss Webster mice were engrafted with a defined microbial consortium consisting of 17 human *Clostridia* isolates [C17; (41–43)] or C17 and *D. piger* ATCC 29098 [*D. piger*; (39, 40)] on 3 consecutive days. Seven days after initial engraftment, mice were infected with 10^9^ CFU of a *S.* Typhimurium *phsA asrA* mutant (*phsA asrA*) or the *S.* Typhimurium WT. Two days after *S.* Typhimurium infection, mice were inoculated with 10^9^ CFU of a 1:1 mixture of *E. coli* Nissle 1917 (WT) and an isogenic *cydA* mutant. (*D*) Schematic showing timing and type of treatment. (*E*) Feces were collected at the indicated timepoints to determine *S.* Typhimurium colonization by enumerating CFU. Data are pooled from two independent experiments (N = 8 for C17 + *phsA asrA*; N = 7 for C17 + WT; N = 15 for C17 + *D. piger* + *phsA asrA*; N = 14 for C17 + *D. piger* + WT). (*F*) Cycle threshold (Ct) values of total *Eubacteria* and *D. piger* 16 s rRNA genes were determined by quantitative real-time PCR using genomic DNA isolated from cecal contents collected 10 d after initial engraftment. (*G*) The CI of *E. coli* wild type versus *cydA* mutant in the feces was determined by enumerating CFU 24 h post administration. The box plots represent the first to third quartiles, and the line indicates the median value. Data are pooled from three independent experiments (N = 8 for C17 + *phsA asrA*; N = 7 for C17 + WT; N = 20 for C17 + *D. piger* + *phsA asrA*,; N = 21 for C17 + *D. piger* + WT). The unpaired *t* test with Welch’s correction (*E* and *G*) or ordinary one-way ANOVA with the Tukey post-test (*A*–*C* and *F*) was performed on logarithmically transformed values. ^∗^*P* < 0.05; ^∗∗^*P* < 0.01; ^∗∗∗^*P* < 0.001.

To determine whether *S.* Typhimurium uses its *phsABC* and *asrABC* operons to directly reduce *cydA*-mediated growth of *E. coli*, we first performed coculture experiments in vitro. Cytochrome *bd* oxidase-mediated aerobic respiration conferred a similar fitness advantage upon *E. coli* when cocultured with the *S.* Typhimurium wild type or a *phsA asrA* mutant (Fig. 4*A*). Next, we wanted to determine whether *Deltaproteobacteria* were necessary for *S.* Typhimurium to inhibit cytochrome *bd* oxidase-mediated aerobic respiration in *E. coli.* Notably, upon addition of *Desulfovibrio piger* (39, 40), a representative of the *Deltaproteobacteria*, *cydA*-mediated growth of *E. coli* was significantly reduced in the presence of the *S.* Typhimurium wild type, but not in the presence of a *S.* Typhimurium *phsA asrA* mutant (Fig. 4*A*). These results suggested that the *phsABC* and *asrABC* operons of *S.* Typhimurium limit *cydA*-mediated respiration in *E. coli* only in the presence of *Deltaproteobacteria*. Interestingly, addition of *D. piger* also reduced growth of *S.* Typhimurium in vitro (Fig. 4*B*). However, when thiosulfate was added to the medium, the *phsABC* and *asrABC* operons enhanced growth of *S.* Typhimurium in the presence of *D. piger* (Fig. 4*C*), suggesting that anaerobic thiosulfate respiration enables the pathogen to overcome growth inhibition by *Deltaproteobacteria.*

Next, we wanted to investigate the role of the *S.* Typhimurium *phsABC* and *asrABC* operons in reducing *cydA*-mediated respiration of *E. coli* in vivo using a model that provided experimental control over the presence of *Deltaproteobacteria.* To this end, germ-free Swiss Webster mice received a defined microbial community consisting of 17 human *Clostridia* isolates (41–43) alone, or in combination with *D. piger via* oral gavage over 3 consecutive days (Fig. 4*D*). Seven days after the initial inoculation, mice were infected with either the *S.* Typhimurium wild type or an isogenic *phsA asrA* mutant (Fig. 4*E*). Two days after *S.* Typhimurium infection, mice were inoculated with a 1:1 mixture of the *E. coli* Nissle 1917 wild type and an isogenic *cydA* mutant. Successful colonization of *D. piger* in recipient mice was confirmed by real-time PCR (Fig. 4*F*). In the absence of *D. piger*, the ability of *S.* Typhimurium to produce sulfide did not reduce the fitness advantage conferred upon *E. coli* by *cydA*-mediated respiration. In contrast, in the presence of *D. piger*, the *cydA*-mediated fitness advantage in *E. coli* was significantly reduced in mice infected with the *S.* Typhimurium wild type, but not in mice infected with a *S.* Typhimurium mutant unable to produce hydrogen sulfide (*phsA asrA* mutant) (Fig. 4*G*). These results suggested that hydrogen sulfide production by *S.* Typhimurium limits *cydA*-mediated respiration in *E. coli* only in the presence of *Deltaproteobacteria*.

## Discussion

The gut microbiota confers colonization resistance against *S.* Typhimurium (44), which relies crucially on competition for resources. Competition arises between the pathogen and closely related species or taxonomically distinct species that rely on the same resources. Colonization resistance increases with community diversity, which rests critically upon the presence of commensal *E. coli,* a close relative consuming resources that exhibit considerable overlap with those used by *S.* Typhimurium (25, 38, 45, 46).

To overcome colonization resistance, *S.* Typhimurium uses its virulence factors to trigger intestinal inflammation (47), which is accompanied by increased diffusion of oxygen into the lumen of the large intestine (21, 22). The gut microbiota is dominated by obligately anaerobic *Clostridia* and *Bacteroidia* species, which do not compete with the pathogen for oxygen. However, competition for this resource arises between the pathogen and closely related minority species, such as commensal *E. coli* (25, 45). Here, we show that functional *phsABC* and *asrABC* operons enable *S.* Typhimurium to gain an edge during this competition by blocking aerobic respiration of the commensal. These data suggest that hydrogen sulfide production is part of a business plan that enables *Salmonella* serovars to overcome competition with closely related species present in the gut microbiota. Consistent with this idea, the *phsABC* and *asrABC* operons were only required for *S.* Typhimurium growth in the presence of a complex gut microbiota that harbored commensal *E. coli*.

Inhibition of aerobic respiration in *E. coli* by the *S.* Typhimurium *phsABC* and *asrABC* operons was dependent on the presence of *Deltaproteobacteria*, a group of obligately anaerobic bacteria that produce hydrogen sulfide by using thiosulfate or taurine as terminal respiratory electron acceptors (37). The basis for the polymicrobial synergy between *S.* Typhimurium and *Deltaproteobacteria* remains unclear but it is possible that cumulative production of hydrogen sulfide by both taxa raises concentrations of this volatile compound above a threshold needed to inhibit cytochrome *bd* oxidase activity in *E. coli* (48). This concept is consistent with the finding that an elevated abundance of *Deltaproteobacteria* inhibits cytochrome *bd* oxidase-mediated aerobic respiration of *C. rodentium* in the murine cecum (18). However, hydrogen sulfide is notoriously difficult to measure in vivo (49), which prevented us from testing this hypothesis directly.

In summary, our study reveals that the ability to produce hydrogen sulfide enables *S.* Typhimurium to gain an edge during its competition with close relatives, such as *E. coli*, and maintain a high pathogen abundance in the feces (*SI Appendix*, Fig. S1). A high *S.* Typhimurium burden in the feces is required for transmission by the fecal oral route (21, 50), which is the principal driving force of natural selection acting on an enteric pathogen that circulates in animal reservoirs. Whereas hydrogen sulfide production has been used for almost a century to distinguish *Salmonella* serovars from *E. coli* or *Shigella* species (1–3), our findings finally help explain why this biochemical trait is conserved in the genus *Salmonella*.

## Materials and Methods

### Ethics Statement.

All mice used in the study were maintained under germ-free or specific pathogen-free conditions in the Animal Association of Laboratory Animal Care-accredited University of California, Davis, Teaching and Research Animal Services. All protocols were approved by the Institutional Animal Care and Use Committee at the University of California, Davis. Animals were euthanized by CO_2_ asphyxiation followed by cervical dislocation according to NIH Animal Research Advisory Committee (ARAC) Guidelines.

### Bacterial Strains and Growth Conditions.

*E. coli* and *S.* Typhimurium strains used in this study are listed in *SI Appendix*, Table S1. *E. coli* and *S.* Typhimurium strains were cultured in Luria-Bertani (LB) broth (LB, BD Biosciences) or on LB agar plates. Broth culture and plate incubation was carried out under atmospheric conditions at 37 °C for 16 to 20 h (33). Human *Clostridia* isolates were grown anaerobically in Gifu Anaerobic Medium Broth (HiMedia) for at least 72 h. *D. piger* was grown for 72 h anaerobically in Tripticase Soy Broth supplemented with 5% sheep blood Agar plates and liquid media were supplemented with antibiotics used at the following concentrations when required: carbenicillin (Carb), 100 μg/mL; chloramphenicol (Cm), 30 μg/mL; Nalidixic acid (Nal), 50 μg/mL; and kanamycin (Kan), 100 μg/mL.

### Strain Construction.

Mutants in *S.* Typhimurium strain AJB715 were constructed by deleting the coding sequences of *asrA, phsA,* and *ttrABC* using primers and plasmids listed in *SI Appendix*, Tables S2 and S3, respectively. Briefly, primers were designed to amplify approximately 500 base pair DNA regions located upstream and downstream of the coding region. The resulting PCR product was inserted into plasmid pRDH10 digested with BamHI in accordance with the NEBuilder HiFi DNA Assembly protocol (New England Biolabs). Mutant alleles were integrated onto the chromosome of AJB715 as described previously (51). Briefly, pRDH10-derivatives containing the in-frame deletion and gene-specific flanking regions were introduced into *S.* Typhimurium by conjugation with *E. coli* S17-1λpir. Colonies carrying pRDH10-derivatives integrated by homologous recombination into the *S.* Typhimurium chromosome were selected with chloramphenicol and kanamycin, then grown overnight on LB agar without selection to allow for recombination. Dilutions of *S.* Typhimurium were plated on LB with 8% sucrose for counterselection (loss of plasmid). Deletion strains were confirmed through PCR and characterized for their ability to reduce thiosulfate/sulfide by inoculating bismuth sulfite agar plates (BD) and triple sugar iron agar (BD) slants.

### Animal Experiments.

Gnotobiotic Swiss Webster mice were purchased from Taconic Farms and bred at the UC Davis Genome and Biomedical Sciences Facility, maintained by investigators and fed Teklad irradiated 2,918 diet containing 18.4% protein and 6% fat. Female 5 to 7 wk old CBA/J mice were purchased from The Jackson Laboratories. Upon arrival, CBA/J mice were maintained on 5058-PicoLab Mouse Diet 20 (Lab Diet), an irradiated diet containing 20% protein and 9% fat. Animals were left undisturbed for at least a week to allow for acclimation to the vivarium. Mice were confirmed to be *Enterobacterales* negative by plating fecal homogenates on McConkey agar. *S.* Typhimurium infections were performed by delivering 1 × 10^9^ CFU/mouse in a 0.1 mL volume of LB broth intragastrically by oral gavage. Mock infections were performed by delivering 0.1 mL of sterile LB broth intragastrically. For experiments using a consortium of human *Clostridia* isolates and *D. piger*, each *Clostridia* strain was grown individually for 72 h anaerobically in Gifu Anaerobic Medium broth, and *D. piger* was grown for 72 h anaerobically in Trypticase Soy Broth (BD) supplemented with 5% sheep blood (Thermo Fisher Scientific). Equal volumes of each culture were combined and used to gavage mice orally with 0.2 mL of the resulting mixture. The fecal lipocalin-2 concentration was determined by collecting freshly voided fecal pellets in 2 mL centrifuge tubes at the indicated time points. At the end of the experiment, animals were euthanized by CO_2_ asphyxiation followed by cervical dislocation.

The bacterial burden was determined by collecting freshly voided fecal pellets in 2 mL centrifuge tubes at the indicated time points, as well as by collecting cecal content in 2 mL centrifuge tubes and liver in 5 mL tissue homogenizer tubes containing 1 mL phosphate-buffered saline (PBS), 0.9 to 2.00 mm round and 5.6 mm cone-shaped stainless-steel beads (NextAdvance) after euthanasia performed following NIH ARAC Guidelines. Tissues were kept on ice before being homogenized using a Vortex Genie 2 (Scientific Industries) equipped with a vertical microtube holder (Scientific Industries), or a Bullet Blender Storm 5 (NextAdvance). Homogenization was carried out at the maximum vortex intensity for 5 min or until complete homogenization of samples was achieved. Series 10-fold dilutions of samples were plated in on LB agar plates (BD Biosciences) containing the appropriate antibiotics for selection. The CI was calculated by enumerating wild type and mutant *E. coli*, determining the ratio in the samples, and then dividing by the input ratio determined in the inoculum.

### Quantification of FGF15 Serum Levels by ELISA.

For FGF15 quantification in the serum, blood was collected at necropsy by cardiac puncture using a 30G needle and then incubated at room temperature for 1 h prior to centrifugation for 15 min at 1,500×*g* at 4 °C to pellet red blood cells. Then, the serum was collected, aliquoted, and stored at −20 °C until analysis. FGF15 levels were determined in the serum using the FGF-15 ELISA kit (Biomatik) according to the manufacturer’s protocol.

### Quantification of Fecal Lipocalin-2 by ELISA.

Freshly collected fecal samples were resuspended in PBS and homogenized using a Vortex Genie 2 (Scientific Industries) equipped with a vertical microtube holder (Scientific Industries), at maximum vortex intensity for 5 min or until complete homogenization of samples was achieved. These samples were then centrifuged for 10 min at 10,000×*g* and 4 °C. Supernatants were collected and stored at −20 °C until analysis. Lipocalin-2 levels were estimated in the supernatants using the Duoset murine Lipocalin-2 ELISA kit (R&D Systems) according to the manufacturer’s protocol.

### Isolation of Ileal Epithelium.

Epithelial cells were isolated from freshly collected ileal tissue, defined as the last 10 cm of the small intestine immediately proximal to the cecum (33). Ileal tissue was cut longitudinally to expose the epithelial surface and then cut laterally into several pieces before being placed into ice-cold PBS containing 0.5 M ethylenediaminetetraacetic acid (EDTA) and 1.5 mM dithiothreitol (DTT) for 20 min. Ileal tissue was then moved to PBS containing 0.5 M EDTA and incubated at 37 °C for 10 min. Tubes containing ileal tissue were vigorously shaken by hand for approximately 30 s to release epithelial cells from the underlying tissue. Intact tissue was removed, and the resulting suspension of epithelial cells was centrifuged at 800×*g* for 5 min at 4 °C. The supernatant was aspirated, and the cell pellet was either transferred to a 2 mL screw cap microtube containing 1 mL of TRI Reagent (Molecular Research Center) for RNA extraction, or to a 5 mL tissue homogenizer tube containing 1 mL ice-cold PBS, UFO, and stainless-steel beads (NextAdvance) for intracellular bile acid concentration measurement.

### Quantification of Bile Acids in Ileal Epithelial Cells, and Ileal and Cecal Contents.

Bile acids in ileal and cecal contents were quantified by LC–MS as described previously (52). Briefly, 15 mg of tissue was placed into 2 mL tubes and 10 μL of bile acid surrogate standards were added. Bile acids were extracted using 500 μL cold methanol and stainless steel grinding balls, in GenoGrinder. Supernatant was transferred to a new Eppendorf tube containing 10 μL 20% glycerol solution in methanol. A second aliquot of 500 μL of cold methanol was added to the centrifugation pellet. This was homogenized again using GenoGrinder, centrifuged, and the second supernatant was combined with the first one. Samples were transferred to Speed-vac and evaporated to dryness. Dry samples were resuspended for LC–MS in 1-phenyl-ureido3-hexanoic acid (PUHA)/1-cyclohexyl ureido dodecanoic acid (CUDA) 50 nM in methanol/Acetonitrile 50:50 by sonication for 5 min. Samples were set on wet ice for 15 min. Then, samples were centrifuged for 3 min at max speed, and supernatant was transferred to glass insert in amber HPLC vial and stored at −20 °C until LC–MS analysis. Analysis was performed on Thermo Scientific Vanquish UPLC/Sciex Qtrap with the targeted MRM method.

Isolated murine ileal epithelial cells were lysed in 1 mL PBS using a Bullet Blender Storm 5 (NextAdvance). Homogenate was transferred to a 1.5 mL Eppendorf tube and centrifuged at 10,000×*g* for 10 min at 4 °C. Supernatant was collected and stored at −80 °C until analysis. Total bile acid levels in the supernatants were measured by colorimetric assay using Total Bile Acid Assay Kit (Cell Biolabs, Inc.) according to the manufacturer’s protocol.

### RNA isolation and qRT-qPCR.

Murine cecal and liver tissues were collected in a 2 mL screw cap microtube and snap-frozen in liquid nitrogen. RNA was isolated from murine enterocytes, cecal and liver tissues using Tri-reagent (Molecular Research Center) according to the manufacturer’s protocol. RNA was resuspended in DNAse buffer and contaminating DNA was removed using the DNA-free kit (Applied Biosystems); RNA concentration and quality were measured spectrophotometrically using a Nandrop (ND-1000, Nanodrop Technologies), and RNA was stored at −80 °C.

Isolated RNA from murine samples was reverse transcribed to cDNA using random hexamers and MuLV reverse transcriptase (Applied Biosystems). Quantitative real-time PCR was performed using SYBR green PCR mix (Applied Biosystems) and the primer sets listed in *SI Appendix*, Table S4 to a concentration of 0.25 mM on a ViiA 7 RT-PCR system (Applied Biosystems). QuantiStudio Real-Time PCR software v1.3 (Applied Biosystems) and the comparative threshold cycle number (Ct) method (2^−ΔΔCt^) were used to calculate fold-changes between experimental groups as follows: ∆∆Ct = (Ct _target mRNA_ – Ct _internal control_)_condition of interest_ – (Ct _target mRNA_ – Ct _internal control_)_control condition_, and the final data were derived from 2^−∆∆CT^.

### Construction of Plasmid for Absolute Quantification of *Deltaproteobacteria* by qPCR.

Plasmids were constructed for absolute quantification of *Eubacteria* and *Deltaproteobacteria*. For *Eubacteria*, **Rectilius* productus* [ATCC 27340D] was used as a reference strain, and its 16S rRNA gene cloned into pCR2.1 TOPO vector was used to determine gene copy number, as previously described in ref. 17. For *Deltaproteobacteria*, *D. piger* was used as a reference strain. Universal primer pairs for the 16S rDNA gene of *Deltaproteobacteria* were selected from literature (53). Bacterial DNA was extracted, and 16s rDNA fragments were amplified by PCR using primers listed in *SI Appendix*, Table S4. PCR products were purified by migration in 1% agarose gel and DNA recovery using the ZymoClean Gel DNA Recovery Kit (Zymo Research) according to the manufacturer’s protocol. The purified PCR product was cloned into pCR II-TOPO to create plasmids listed in *SI Appendix*, Table S3. These plasmids were transformed into chemically competent *E. coli* TOP10 using the TOPO-TA Cloning Kit (Invitrogen) according to the manufacturer’s protocol. After overnight growth in LB broth supplemented with 50 µg/mL kanamycin, plasmids were purified using the QIAprep Spin Miniprep Kit (Qiagen). Plasmid concentration and quality were measured spectrophotometrically using a Nandrop (ND-1000, Nanodrop Technologies) to determine plasmid copy numbers, and were stored at −20 °C.

### Quantification of Fecal Microorganisms by qPCR.

Cecal contents were collected in a 2 mL screw cap microtube and frozen at −20 °C. DNA was extracted using the QIAamp® PowerFecal® Pro DNA Kit (Qiagen) according to the manufacturer instructions, with the following modifications: a) Solution C5 was incubated for 5 min onto the column and b) one wash step with 500 μL 80% ethanol was added as a final wash step to improve DNA purity. Concentration and quality were measured spectrophotometrically using a Nandrop (ND-1000, Nanodrop Technologies). Total bacterial DNA extracted from cecal contents was diluted to 2,5 ng/μL with nuclease-free water (Ambion). To quantify cecal *Eubacteria* and *Deltaproteobacteria*, serial dilutions of plasmids from 1 × 10^9^ copies to 1 × 10^3^ copies per μl, and nontemplate controls were included on each plate to generate an absolute standard curve. To confirm the engraftment of Swiss Webster mice with *D. piger*, serial dilutions of genomic DNA extracted from a pure culture of *D. piger* and nontemplate controls were included on each plate to generate a calibration curve, verify the purity of the amplified product and determine the limit of detection by analyzing the melting curve performed at the end of amplification. Quantitative real-time PCR was performed using SYBR green PCR mix (Applied Biosystems) and the primer sets listed in *SI Appendix*, Table S4 to a concentration of 0.25 mM on a ViiA 7 RT-PCR system (Applied Biosystems). Results were analyzed using QuantiStudio Real-Time PCR software v1.3 (Applied Biosystems). Comparison of Ct of each sample with Ct of standard curves and melt curve profiles were used to determine the absolute quantity of *Eubacteria* and *Deltaproteobacteria*, while comparison of Ct between mouse groups, pure DNA dilutions and melt curve profiles were used to determine the limit of detection and confirm engraftment of *D. piger* in germ-free mice.

### Inhibition of *E. coli* Aerobic Respiration by Hydrogen Sulfide.

LB broth (LB, BD Biosciences) containing 1% calcium carbonate (Sigma) was inoculated with 10^3^ CFU of a 1:1 mixture of the *E. coli* Nissle 1917 wild type and an isogenic c*ydA* mutant, in presence or absence of 1 mM of sodium hydrosulfide (NaHS; Sigma). Broth culture was carried out in standing test tubes, under atmospheric conditions at 37 °C for 14 h. The CI between the two *E. coli* strains was assessed by plating serial dilutions on LB agar containing corresponding antibiotics.

### Competition between *D. piger, E. coli,* and *S*. Typhimurium in vitro.

To assess whether *D. piger* inhibits aerobic respiration of Enterobacterales, and whether *S*. Typhimurium can overcome this inhibition by respiring thiosulfate, trypticase soy broth supplemented with 5% sheep blood, 1% calcium carbonate, and 3% sodium thiosulfate (Sigma) (17) was inoculated with 200 μL of a *D. piger* culture or sterile media, 10^3^ CFU of a 1:1 mixture of the *E. coli* Nissle 1917 wild type and an isogenic c*ydA* mutant, and 10^3^ CFU of *S.* Typhimurium wild type, *S*. Typhimurium *phsA asrA* mutant. Broth culture was carried out under atmospheric conditions at 37 °C for 14 h. The CFU of *E. coli* and *S*. Typhimurium strains was assessed by plating serial dilutions on LB agar containing corresponding antibiotics.

### Statistical Analysis.

An unpaired *t* test with Welch’s correction was performed on logarithmically transformed values when comparing two groups. Ordinary one-way ANOVA with the Tukey post hoc test were performed on logarithmically transformed values when comparing three groups or more.

## Supplementary Material

Appendix 01 (PDF)

Dataset S01 (XLSX)

## Data Availability

All study data are included in the article and/or supporting information.
