# Supplementary material for: Salmonella produces sulfide to compete with Escherichia coli in the gut lumen
Source: Proc Natl Acad Sci U S A. 2025 Sep 12;122(37):e2504095122. doi: 10.1073/pnas.2504095122 (PMC12452934; doi:10.1073/pnas.2504095122)
Supplement: Supplementary file 1 — Appendix 01 (PDF) [file pnas.2504095122.sapp.pdf]

SUPPLEMENTAL FIGURES

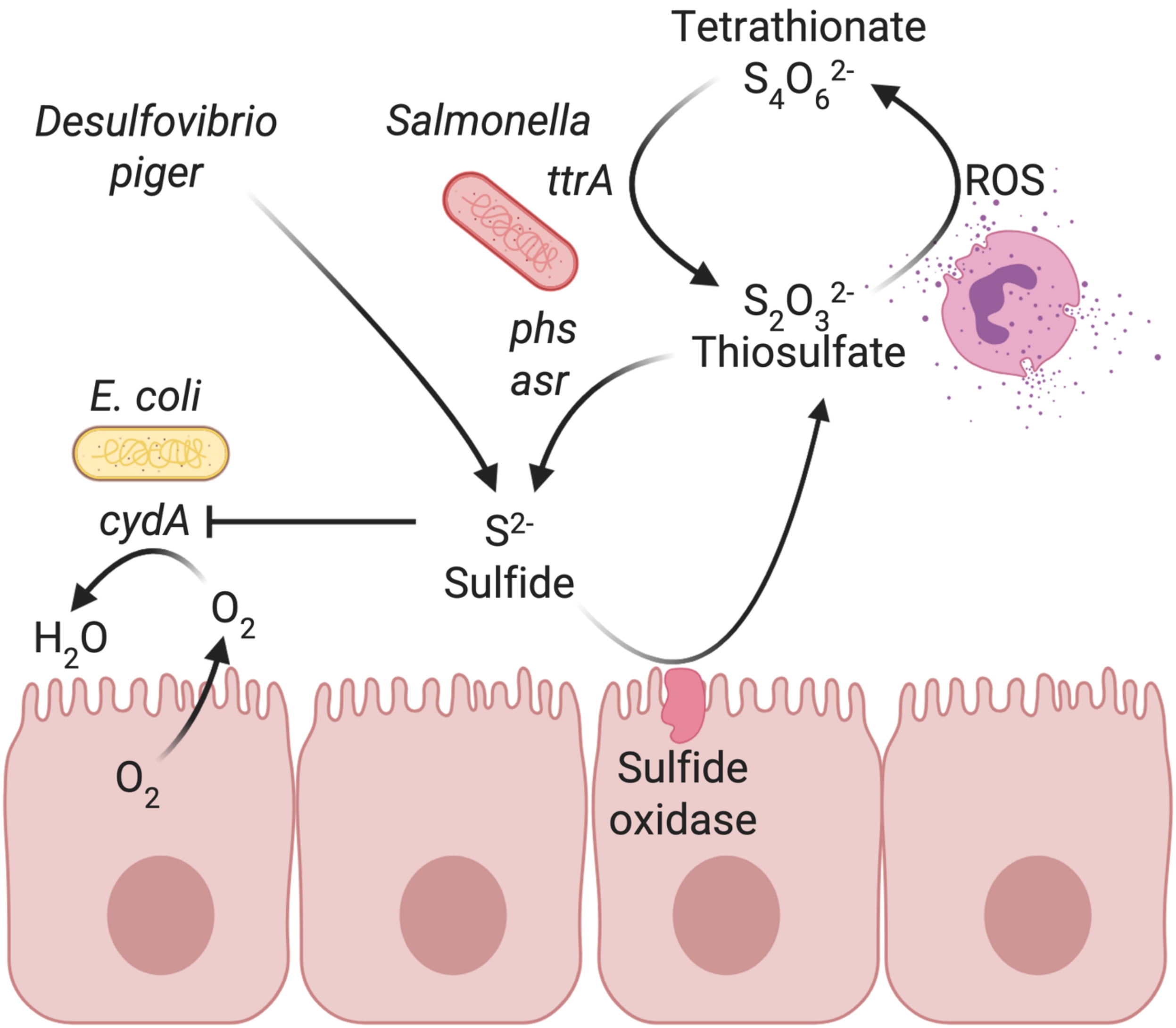

Supplementary Figure 1: Graphical abstract illustrating the proposed mechanism by which hydrogen sulfide production enables *S.* Typhimurium to gain an edge during its competition with close relatives, such as *E. coli*. Created with BioRender.

SUPPLEMENTAL TABLES

Supplementary Table 1: Bacterial strains used in this study.

Bacterial strains		
**Species and strain**	**Genotype**	**Reference**
E.coli TOP10	F- *mcrA* Δ(*mrr-hsdRMS-mcrBC*) Φ80*lacZ*ΔM15 *lacX74 recA 1 araD139* Δ(*ara - leu*)*7697 galU galK rpsL endA1 nupG*	Invitrogen
E. coli S17-1λpir	*E. coli* zxx::RP4 2-(Tet^{R}::Mu) (Kan^{R}::Tn7) *λpir recA1 thi pro hsdR (*r^{-} m^{+}*)*	(1)
D. piger ATCC29098	Wild type isolate	(2, 3)
S. Typhimurium AJB715	ATCC14028 Nal^{R} *phoN*::Km (Nal^{R}, Kan^{R})	(4)
S. Typhimurium FF183	ATCC14028 Nal^{R} *ΔinvA ΔspiB phoN*::Tn10dCam (Nal^{R}, Cm^{R})	(5)
S. Typhimurium AL5	ATCC14028 Nal^{R} *phoN*::Km *ΔasrA* (Nal^{R}, Kan^{R})	This study
S. Typhimurium AL6	ATCC14028 Nal^{R} *phoN*::Km *ΔphsA* (Nal^{R}, Kan^{R})	This study
S. Typhimurium AL7	ATCC14028 Nal^{R} *phoN*::Km *ΔttrABC* (Nal^{R}, Kan^{R})	This study

S. Typhimurium AL8	ATCC14028 Nal^{R} *phoN*::Km *ΔasrA ΔphsA* (Nal^{R}, Kan^{R})		This study
E. coli Nissle 1917 wild-type strain (O6:K5:H1)	Wild type isolate		(6, 7)
YL11	*E. coli* Nissle 1917 *ΔcydA*		(8)
**Consortium of 17 human *Clostridia* and *Erysipelotrichia* isolates**			
**Strain number**	**Old Nomenclature**	**Updated Nomenclature**	**Reference**
St1	*Clostridium saccharogumia*	*Erysipelatoclostridium saccharogumia*	(9-12)
St3	*Lachnospiraceae_7_1_58* FAA	*Flavonifractor plautii*	
St4	*Clostridium hathewayi*	*Hungatella hathewayi*	
St6	*Blautia producta*	*Blautia producta*	
St7	*Clostridium bolteae*	*Enterocloster bolteae*	
St8	*Clostridiaceae* JC13	*Dielma fastidiosa*	
St9	*Clostridium indolis*	*Anaerostipes caccae*	
St13	*Anaerotruncus colihominis*	*Anaerotruncus colihominis*	
St14	*Ruminococcus* sp. ID8	Unclassified *Lachnospiraceae*	

St15	*Clostridium asparagiforme*	*Enterocloster asparagiformis*	
St16	*Clostridium_7_3_54FAA*	*Lachnoclostridium symbiosum*	
St18	*Clostridium ramosum*	*Erysipelatoclostridium ramosum*	
St21	*Eubacterium fissicatena*	*Faecalicatena fissicatena*	
St26	*Clostridium scindens*	*Lachnoclostridium scindens*	
St27	*Lachnospiraceae_3_1_57* FAA	*Eisenbergiella massiliensis*	
St28	*Clostridiales_1_7_47FAA*	*Enterocloster* spp.	
St29	*Lachnospiraceae_3_1_57* FAA	*Eisenbergiella tayi*	

Supplementary Table 2: Primers used for targeted mutagenesis is this study.

Primers used for targeted mutagenesis			
**Target**		**Sequence (5'-3')**	**Reference**
asrA	asrA_AB_fwd	CACACCCGTCCTGTGCCTTGACTCAACGTTTTTATC	This study
	asrA_AB_rev	ACATGCGTTACATCACATACTCCACAGTAATATTC	This study
	asrA_BC_2_fwd	ATGTGATGTAACGCATGTCACATTGTTC	This study
	asrA_BC_2_rev	CCGGCGTAGAGGATCATTTCTTCTTTGTACAGCAC	This study
phsA	phsA_AB_fwd	CACACCCGTCCTGTGTTCTTTCAGAAAGTGTCC	This study
	phsA_AB_rev	TCCTCCCTCACATATAACCTCCTGTTATTAGATATAG	This study
	phsA_CD_fwd	GTTATATGTGAGGGAGGAGGGAACCATG	This study
	phsA_CD_rev	CCGGCGTAGAGGATCGACCCAGCGCTGGATCTC	This study
ttrABC	ttrABC_AB_fwd	CACACCCGTCCTGTGCCGCTTTCCCATCGCGCG	This study
	ttrABC_AB_rev	GGGGGTTACATTGCCAACAATGAAACATTTGTCACGATG	This study
	ttrABC_CD_fwd	GGCAATGTAACCCCCGGGCCGGAGA	This study
	ttrABC_CD_rev	CCGGCGTAGAGGATCGGTTTAAAATCCCTCCGCCATAGCAAC	This study

Supplementary Table 3: Plasmids used in this study.

Name	Genotype	Reference
pRDH10	ori(R6K) *mobRP4 sacRB* Tet^R Cm^R	(5)
pCR ™ II-TOPO ®	Cloning vector, LacZ, Kan^R, Amp^R	Invitrogen
pAL1	pRDH10 with upstream and downstream regions of *S*. Typhimurium *asrA* forming a deletion mutant allele	This study
pAL2	pRDH10 with upstream and downstream regions of *S*. Typhimurium *phsA* forming a deletion mutant allele	This study
pAL3	pRDH10 with upstream and downstream regions of *S*. Typhimurium *ttrABC* forming a deletion mutant allele	This study
pAL4	pCR ™ II-TOPO ® with 16S of *Deltaproteobacteria: D. piger* [ATCC29098]	This study
pSW191	pCR ™ 2.1-TOPO ® with 16S of *Eubacteria: R. productus* [ATCC 27340D]	(13)
pCAL61	pWSK129 with streptomycin resistance cassette (Ω) from pHP45Ω, $Strep^R$, $Spec^R$, Kan^R	(14)
pCAL62	pWSK29 with streptomycin resistance cassette (Ω) from pHP45Ω, $Carb^R$, $Strep^R$	(14)

Supplementary Table 4: Primers used for qPCR and qRT-PCR in This study.

Primers used for quantitative PCR and qRT-PCR			
**Organism**	**Target**	**Sequence (5'-3')**	**Reference**
Bacteria	*Deltaproteobacteria* 16S-Fwd	GGTGTAGGAGTGAARTCCGT	(15)
	Deltaproteobacteria 16S-Rev	TACGTGTGTAGCCCTRGRC	
	Eubacteria 16S-Fwd	ACTCCTACGGGAGGCAGCAGT	
	Eubacteria 16S-Rev	ATTACCGCGGCTGCTGGC	
	D. piger 16S-Fwd	CTACCAAGGCAACGATGGGT	This study
	D. piger 16S-Rev	GAGTTAGCCGGTGCTTCCTT	
Mus musculus	*Cxcl9*-Fwd	TCGGACTTCACTCCAACACA	This study
	Cxcl9-Rev	CCTTATCACTAGGGTTCCTCGAA	
	Cxcl1-Fwd	TGCACCCAAACCGAAGTCAT	(16)
	Cxcl1-Rev	TTGTCAGAAGCCAGCGTTCAC	
	Il17a-Fwd	GCTCCAGAAGGCCCTCAGA	(17)
	Il17a-Rev	AGCTTTCCCTCCGCATTGA	
	Nos2-Fwd	TTGGGTCTTGTTCACTCCACGG	(16)
	Nos2-Rev	CCTCTTTCAGGTCACTTTGGTAGG	
	Fxr (Nr1h4)-Fwd	GCTAATGAGGACGACAGCGA	This study

	Fxr (Nr1h4)-Rev	GAGTTCCGTTTTCTCCCTGC	
	Shp (Nr0b2)-Fwd	GATCCTCTTCAACCCAGATGTG C	This study
	Shp (Nr0b2)-Rev	CTACCAGAAGGGTGCCTGGA	
	Fgf15-Fwd	GCAAGATATACGGGCTGATTCG	This study
	Fgf15-Rev	AGAAGGAGCGGTGAAACACG	
	Cyp7a1-Fwd	TCTACCCAGACCCTTTGACTT	This study
	Cyp7a1-Rev	AACGCTCAGCAGTCGTTACA	
	Cyp8b1-Fwd	CTCCCCATAAGACGCCATCC	This study
	Cyp8b1-Rev	TGAGTCAAGTGTGGGTGAGC	
	Cyp27a1-Fwd	TCTGGCTACCTGCACTTCCT	(18)
	Cyp27a1-Rev	GTGTGTTGGATGTCGTGTCC	
	Cyp7b1-Fwd	TAGGCATGACGATCCTGAAA	(18)
	Cyp7b1-Rev	TCTCTGGTGAAGTGGACTGAAA	
	Gapdh-Fwd	TGTAGACCATGTAGTTGAGGTCA	(17)
	Gapdh-Rev	AGGTCGGTGTGAACGGATTTG	

Supplemental References

1. Simon R, Priefer U, & Pühler A (1983) A Broad Host Range Mobilization System for In Vivo Genetic Engineering: Transposon Mutagenesis in Gram Negative Bacteria. *Bio/Technology* 1(9):784-791.
2. Loubinoux J*, et al.* (2002) Reclassification of the only species of the genus Desulfomonas, Desulfomonas pigra, as Desulfovibrio piger comb. nov. *Int J Syst Evol Microbiol* 52(Pt 4):1305-1308.
3. Moore WEC, Johnson JL, & Holdeman LV (1976) Emendation of Bacteroidaceae and Butyrivibrio and Descriptions of Desulfomonas gen. nov. and Ten New Species in the Genera Desulfomonas, Butyrivibrio, Eubacterium, Clostridium, and Ruminococcus. *International Journal of Systematic Bacteriology* 26(2):238-252.
4. Kingsley RA*, et al.* (2003) Molecular and phenotypic analysis of the CS54 island of Salmonella enterica serotype typhimurium: identification of intestinal colonization and persistence determinants. *Infect Immun* 71(2):629-640.
5. Faber F*, et al.* (2016) Host-mediated sugar oxidation promotes post-antibiotic pathogen expansion. *Nature* 534(7609):697-699.
6. Grozdanov L*, et al.* (2004) Analysis of the genome structure of the nonpathogenic probiotic Escherichia coli strain Nissle 1917. *J Bacteriol* 186(16):5432-5441.
7. Nissle A (1925) Weiteres über grundlagen und praxis der mutaflorbehandlung. *DMW-Deutsche Medizinische Wochenschrift* 51(44):1809-1813.

8. Litvak Y*, et al.* (2019) Commensal Enterobacteriaceae Protect against Salmonella Colonization through Oxygen Competition. *Cell Host Microbe* 25(1):128-139 e125.

9. Atarashi K*, et al.* (2013) Treg induction by a rationally selected mixture of Clostridia strains from the human microbiota. *Nature* 500(7461):232-236.

10. Narushima S*, et al.* (2014) Characterization of the 17 strains of regulatory T cell-inducing human-derived Clostridia. *Gut Microbes* 5(3):333-339.

11. Atarashi K*, et al.* (2011) Induction of colonic regulatory T cells by indigenous Clostridium species. *Science* 331(6015):337-341.

12. Tiffany CR*, et al.* (2021) The metabolic footprint of Clostridia and Erysipelotrichia reveals their role in depleting sugar alcohols in the cecum. *Microbiome* 9(1):174.

13. Winter SE*, et al.* (2010) Gut inflammation provides a respiratory electron acceptor for Salmonella. *Nature* 467(7314):426-429.

14. Spees AM*, et al.* (2013) Streptomycin-induced inflammation enhances Escherichia coli gut colonization through nitrate respiration. *mBio* 4(4).

15. Tirelle P*, et al.* (2020) Comparison of different modes of antibiotic delivery on gut microbiota depletion efficiency and body composition in mouse. *BMC Microbiol* 20(1):340.

16. Godinez I*, et al.* (2008) T cells help to amplify inflammatory responses induced by Salmonella enterica serotype Typhimurium in the intestinal mucosa. *Infect Immun* 76(5):2008-2017.

17. Overbergh L*, et al.* (2003) The use of real-time reverse transcriptase PCR for the quantification of cytokine gene expression. *J Biomol Tech* 14(1):33-43.

18. Lefort C, Van Hul M, Delzenne NM, Everard A, & Cani PD (2019) Hepatic MyD88 regulates liver inflammation by altering synthesis of oxysterols. *Am J Physiol Endocrinol Metab* 317(1):E99-E108.
